# Collaboration and Age-Friendly Integration – The Development of the FlourishCare Centers of Excellence

**DOI:** 10.1093/geroni/igaf122.062

**Published:** 2025-12-31

**Authors:** Barbara Gordon, Anna Faul, Pamela Yankeelov, Sam Cotton

**Affiliations:** University of Louisville, Louisville, Kentucky, United States; University of Louisville, Louisville, Kentucky, United States; University of Louisville, Louisville, Kentucky, United States; University of Louisville, Louisville, Kentucky, United States

## Abstract

As part of our Geriatric Workforce Enhancement Program, Kentucky developed statewide FlourishCare Centers of Excellence (FC-CEs), bringing together experts from universities, healthcare providers, community organizations, and local governments to improve how care is provided to older adults. The goal is to make sure healthcare professionals have the specialized knowledge needed to support aging adults in all areas of care. FlourishCare Centers of Excellence works closely with community partners to ensure older adults can easily access personalized services that help them stay independent, maintain their dignity, and improve their overall well-being. By connecting different systems—such as hospitals, nursing homes, senior care programs, and government services—we created a smooth and coordinated approach to healthcare. FC-CEs ensures that older adults, including those with dementia, receive continuous and well-organized age-friendly care and support, whether they are at home, in a hospital, or in a care facility. These centers are led by Area Agencies on Aging (AAAs), which serve as regional coordinators to ensure seamless collaboration between healthcare and community systems. A dedicated steering committee guides the initiative’s strategic direction. This session will explore the successes of the FC-CE initiative, including the integration of age-friendly principles in primary care and the advancement of workforce development programs. Attendees will learn about effective partnership models, strategies for replication in other regions, and solutions to challenges faced during implementation. FC-CEs are setting a new standard for age- and dementia-friendly care, ensuring that older adults receive high-quality, coordinated, and compassionate services across all care settings.

